# Experiences of Help-Seeking for Severe Mental Health Problems in Young Pakistani Women: A Preliminary Qualitative Study

**DOI:** 10.1177/00220221241236944

**Published:** 2024-05-01

**Authors:** Chiara Causier, Louise Johns, Jerica Radez, Hibah Hassan, Daniel Maughan, Felicity Waite

**Affiliations:** 1 University of Oxford, UK; 2 Oxford Health NHS Foundation Trust, UK

**Keywords:** youth mental health, help-seeking, diversity, ethnic minority, access

## Abstract

Almost three quarters of mental illnesses start by the age of 25, yet youth (18–25-year-olds) are often underrepresented in U.K. services. This is particularly true for those of ethnic minorities. In this study, we aimed to understand how young Pakistani women and their parents make decisions to seek help for severe mental health problems, and the barriers and facilitators to accessing professional help. Young Pakistani women with experience of severe mental health problems and their parents were recruited from a community sample. Semi-structured interviews were conducted with six young people and two parents. Data were analyzed using reflexive thematic analysis. Pakistani culture and its interplay with British culture strongly influenced the decisions and ability of young Pakistani women and their parents to help-seek, largely through the role of stigma. Low mental health literacy, stigma, and a lack of culturally informed services were identified as the most common barriers to accessing care. These barriers fed into the internalized stigma these young women experienced which, through fear of damaged reputation and personal prejudices, posed further barriers to seeking help. Participants highlighted recommendations for both individual-level (e.g., increased education and awareness) and service-level (e.g., greater choice over care) change to facilitate accessibility of professional help. Young Pakistani women face multiple culturally related challenges to accessing care for severe mental health problems at both the individual- and service-level. Novel suggestions to address these challenges, such as including youth peer support workers in services, may facilitate more inclusive and accessible services.

## Introduction

The United Kingdom is home to the largest Pakistani community in Europe, with over 1.5 million British Pakistani residents. Almost 9% of this community are young Pakistanis aged between 18 and 24, with this population representing the largest demographic of Pakistanis in the United Kingdom (Office for National Statistics, 2021). Despite this, young Pakistanis are often cited as underrepresented in mental health services (Kapadia et al., 2017). This is problematic given that 62.5% of all mental health problems, and 75% of severe mental health problems (SMHPs) are diagnosed by the age of 25 (Kim-Cohen et al., 2003; Solmi et al., 2021); this age period is therefore important for identifying and addressing SMHPs. SMHPs, also referred to as severe mental illness (NHS England, 2022b) constitute mental, behavioral or emotional disorders that “result in serious functional impairment which interferes with or limits one or more major life activities” (National Institute of Mental Health, 2023). When left untreated, they can have major long-term adverse effects on physical health (Goodman et al., 2011), social relationships (Ford et al., 2013), and future employment (Clayborne et al., 2019). It is crucial, therefore, that timely mental health support is provided to young Pakistanis to reduce the long-term negative outcomes that are associated with SMHPs (McGorry & Mei, 2018; Patalay & Fitzsimons, 2018).

Given the prevalence of mental health difficulties in Pakistan (Mirza & Jenkins, 2004) it would be expected for the Pakistani diaspora within the United Kingdom to show at least similarly high levels of mental health difficulties. Moreover, as migration is associated with increased social challenges such as isolation and racism (Ceri et al., 2017; Close et al., 2016) we may even expect to see increased mental health difficulties in this group. The influence of British colonialism, for example, is prominent in the history and culture of Pakistanis (Sohail et al., 2017). In the United Kingdom, it was only following the turmoil of Pakistan’s independence from the British Empire in 1947, that immigration from Pakistan increased (Niaz & Nasir, 2018), and while many of these immigrants intended temporary relocation, many became permanently displaced following the Commonwealth Immigrants Act (1962) which prohibited further primary immigration (Dahya, 1973). Researchers have proposed that the impacts of the socio-political instability, regional conflict, and displacement caused by British colonialism are still a primary cause of mental health difficulties in Pakistanis today (Sohail et al., 2017). Given socio-political tensions with Britain, it is perhaps understandable that help for mental health difficulties may not be readily sought from the Pakistani community. Greater understanding of the experiences of Pakistanis specifically, and how to reduce barriers to them accessing U.K. services, may not only benefit individuals but may provide avenues to improve societal dynamics more broadly.

Until recently, the majority of research has focused on aggregating multiple ethnic groups into the category “South Asian” (Goel et al., 2023; Moller et al., 2016; Stein et al., 2003), leaving minimal consideration of the unique risk factors and barriers that young Pakistanis may face related to their cultural experiences. Furthermore, the limited research that has focused on barriers to mental help-seeking in Pakistani communities has lacked focus on key intersections, such as the one of ethnicity and sex, which may result in vastly different experiences (Arora et al., 2016; Kapadia et al., 2017; Wales et al., 2017). Pakistani women, for example, are frequently reported to experience more mental health difficulties such as persistent depression (Gater et al., 2009), and higher rates of self-harm (Cooper et al., 2006) when compared with Pakistan men. Suggested reasons for this include higher levels of social isolation, unhappy marriages, inter-generational conflicts (Gask et al., 2011), and lower social mobility (Tabassum et al., 2000), which may be more common in some Pakistani communities due to patriarchal norms. For some young Pakistani women (YPW), the expression of mental illness is thought to impinge upon family honor and self-respect (“izzat”; Gunasinghe et al., 2019), leading to family ostracism, reduced marriage prospects, and increased violence (Sangar & Howe, 2021; Tabassum et al., 2000). Barriers to mental health help-seeking specific to YPW are therefore likely to exist, but these are not currently well understood.

This intersection becomes more nuanced still when considering the impact of migration. For instance, in first-generation YPW migrants, higher levels of settlement challenges have been reported due to rigid gender roles (Ahmad et al., 2005) which could pose additional risk factors for SMHPs. Interestingly, however, due to the presence of dual cultural identities, second-generation YPW migrants may be more likely to participate in British community life (Kapadia et al., 2017) which could reduce some of the impacts such as social isolation. The latter may come with its own challenges, however, with second-generation migrants shown to have higher intergenerational conflicts caused by asymmetric family acculturation (Ceri et al., 2017). These intergenerational conflicts may be of particular interest when thinking of help-seeking in YPW given that parents often have a key role in identifying and seeking help for their child’s mental illness (Boulter & Rickwood, 2013; Murphy et al., 2022). Interestingly, there has been evidence to suggest that the parents of Pakistani young people may be significantly less likely than White British parents to seek professional mental health support for their children (Shah et al., 2004; Stein et al., 2003), however, the perspectives of Pakistani parents surrounding help-seeking for mental health difficulties in their daughters has yet to be explored.

Here, a preliminary interview study was conducted with YPW and their parents to understand the in-depth, personal perspectives of the experiences of mental health help-seeking for YPW in the United Kingdom. A qualitative, rather than a quantitative questionnaire, approach was chosen to capture the nuance of participants’ experiences and to take an inductive approach to data collection and analysis. We aimed to explore how YPW and their parents made decisions about seeking professional help for their/their child’s SMHP and what the perceived barriers and facilitators were to seeking help from mental health services. This study was conducted as part of a quality improvement project in Oxfordshire, where it is estimated that while around 6.6% of the population are Pakistani (Oxfordshire Insight, 2021), (a proportion higher than the national average), this population is often underrepresented in community mental health services (e.g., 3% in Oxfordshire Early Intervention Service). Understanding the reasons for this underrepresentation may allow for the development of more inclusive and accessible mental health services in the United Kingdom.

## Method

### Design

A qualitative interview study using reflexive thematic analysis (Braun & Clarke, 2006, 2019) was conducted, which followed the COREQ Checklist (Tong et al., 2007). Ethical approval was received from the University of Oxford Research Ethics Committee (R81751/RE001).

### Position Statement and Service Context

The study team consisted of clinicians with expertise in psychosis and/or youth mental health, and consisted of members who identified as White British, Eastern European, and Pakistani. Two of the authors (LJ and DM) in this paper worked in OEIS, a mental health service for 14 to 65 year olds experiencing first-episode psychosis, where people of Pakistani backgrounds are underrepresented, accounting for only 3% of referrals despite their representation of 6.6% within Oxfordshire (Oxfordshire Insight, 2021). Data collection and analysis was conducted by CC (White British female trainee clinical psychologist) who also had lived experience of SMHP in adolescence. Given this position, regular reflection with the research team was carried out throughout data collection, analysis and interpretation, to minimize biases based on these prior experiences.

### Public and Patient Involvement

All study materials and recruitment strategies were developed by the research team. Interview guides, information sheets, and consent forms were also developed with three YPW and one parent of a YPW who were not participants in the study, to ensure cultural sensitivity of the questions.

### Participants

Participants were recruited from the general population in Oxfordshire through social media (Twitter, LinkedIn, Facebook), email outreach to charitable organizations (e.g., Oxfordshire Mind, Oxford Against Cutting), community outreach (e.g., community groups, mosques), and posters. Participants were included if they, or in the case of parents, their child, was aged 18 to 25 years (a young adult; Salaheddin & Mason, 2016), identified as a woman, identified as Pakistani, lived in or had a GP in Oxfordshire, and perceived themselves to have experienced an SMHP (defined as anxiety/low mood/psychosis that significantly interferes with everyday functioning). Exclusion criteria were insufficient English for interviews; lack of access to Microsoft Teams; and lack of capacity to consent. All participants were screened with an initial online screening and further eligibility checks were conducted via email and at the start of interviews. Based on this, eight YPW and seven parents were initially screened as eligible however, later eligibility checks identified that one young person did not live in Oxfordshire, and five parents and one young person were imposter participants (Ridge et al., 2023). No participants declined or dropped out. Therefore, six young women and two parents participated in the study.

### Procedure

All eligible participants were emailed the information sheet and invited to a video call with CC (lead researcher). Participants were offered remuneration for their time. At the start of the call participants were asked to provide verbal consent. A digital consent form was signed on each participant’s behalf. Demographic information was collected.

Semi-structured interviews (24–74 minutes, *M* = 49.9, *SD* = 17.1) were conducted by CC via video call. The interview topic guide included open-ended questions about their understanding and personal experience of their/their child’s SMHP, experience of and attitudes to help-seeking, and perceived barriers and facilitators to help-seeking. At the start of all interviews, CC emphasized the research rationale, reasons for her interest in this area, and the importance of the participant’s views to minimize power imbalances and promote engagement. At the end of interviews participants were provided with an opportunity to debrief with the researcher and were emailed again with the information sheet which contained signposting to mental health resources. All interviews were audio-recorded, anonymised, and transcribed verbatim.

### Analysis

Reflexive thematic analysis was used to analyze the transcripts. This approach was chosen given its theoretical flexibility and ability to identify patterns within and across data in relation to participants’ lived experience, which aligns with the study aims (Nowell et al., 2017). An inductive approach was used to establish clear links between the research aims and raw data and was rooted in critical realism to reduce researcher bias. Given the small sample size of parents, YPW and parents were treated as a single group for coding and theme generation. Any specific views of each group are reported within the results, to highlight any differences.

Six phases of analysis were followed (Byrne, 2022) in an iterative process, which was led by CC. Data were familiarized through transcription, reading, and re-reading the data set. An initial set of codes was derived from the data using latent and semantic coding, and these codes were refined, grouped into themes, and further refined through regular discussion with the research team (LJ and FW). For example, the code “wider education and awareness” was initially amended to “wider awareness and raising education,” before being refined to “education on mental health problems and services is powerful.” HH, who identified as a YPW, consulted on the proposed thematic structure with a focus on improving the appropriateness and accessibility of the findings for YPW. NVivo 1.6.2 software (QSR International Pty Ltd., 2022) was used to facilitate analysis.

A reflexive journal was kept by CC, to note ideas that arose from data familiarization and coding for each participant. This was used to help create an awareness of initial patterns across interviews and minimize biases. For example, some codes were noticed to be influenced by a previous thematic analysis CC had conducted on barriers to health care, but through the reflexive process were discerned as less relevant to this population.

## Results

### Demographic Information

Six YPW (*M* = 21.67 years old, range 18–25), and two mothers (*M* = 53 years old, range 52–54) were recruited, all of whom identified as following Islam. Four YPW and one parent were first-generation Pakistani, with the other two participants of second-generation. SMHPs participants described experiencing during interviews were predominantly anxiety disorders and depression. All YPW had sought help from mental health professionals, and one of the two parents had sought professional help on the child’s behalf. All participants had experiences of seeking help in the United Kingdom either directly or through their child, predominantly through academic institutes or the NHS. For some, there were additional experiences of help-seeking privately in their home countries. Participant characteristics are presented in Table 1.

**Table 1.** Participant Characteristics.

Participant	Lived experience of SMHPs^a^	Help-seeking experiences
YP1	Social anxiety, generalized anxiety, low mood	School/university mental health services and healthcare services. All in the United Kingdom. Started around 14 years old.
YP2	Depression, anxiety	School/university mental health services and healthcare services. All in the United Kingdom. Started around 14/15 years old.
YP3	Low mood and anxiety related to physical health condition	Healthcare services. All in the United Kingdom. Started around 12/13 years old.
YP4	Generalized anxiety	Healthcare services in Pakistan. School/university mental health services in United Kingdom. Started around 13/14 years old.
YP5	Depression, anxiety	School/university mental health services. All in the United Kingdom. Started around 19/20 years old.
YP6	Anxiety, low moods	Private services. School/university mental health services. All in the United Kingdom. Started around 14/15 years old.
Parent1	Generalized anxiety, eating difficulties^b^	Supported young person accessing healthcare services in Pakistan, and school/university mental health services in the United Kingdom. Started when their child was around 13/14 years old.
Parent2	Low mood and anxiety related to physical health condition^b^	Directly contacted healthcare services. All in the United Kingdom. Started when their child was around 12/13 years old.

a Participants’ self-described experiences, not diagnosed by researchers.

b Characteristics related to the child they supported to help-seek.

### Overview of Themes

**Figure 1.**
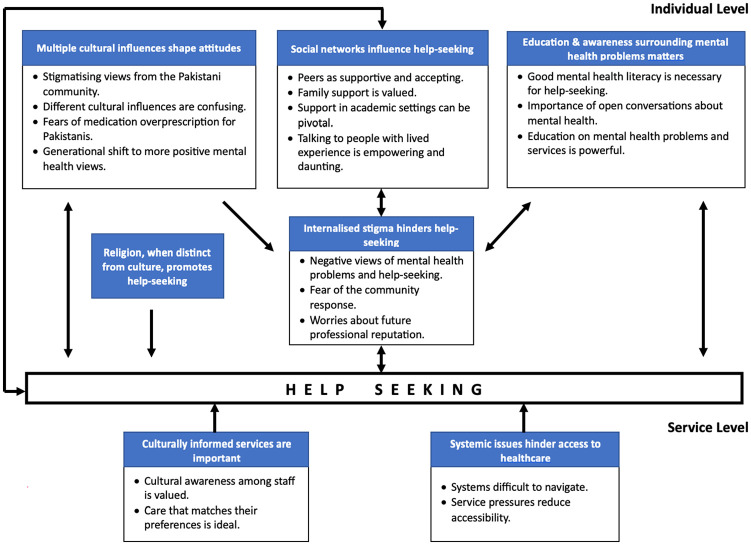
Final Thematic Map.

Seven themes were generated which explored the objective of understanding the experiences of help-seeking for SMHPs in YPW. At the individual level, YPW and their parents discussed how help-seeking was shaped by their personal beliefs and internalized stigma, which was predominantly influenced by their social networks, the impact of growing up with both British and Pakistani cultures, and low mental health literacy (MHL). Religion on the other hand, when distinct from culture, facilitated help-seeking. Both YPW and parents spoke of individual-level implementations that may reduce these individual barriers such as increased education and awareness campaigns. At the service-level, participants (predominantly YPW) spoke of how service pressures could reduce accessibility, and spoke of the value of culturally informed services with greater choice over care in facilitating help-seeking for SMHPs. Themes with outlined quotes are presented below. The final thematic map is presented in Figure 1.

#### Individual Level

##### Multiple Cultural Influences Shape Attitudes

YPW shared that *different cultural influences are confusing*, referring to differences between British and Pakistani cultures. Participants voiced that in Pakistan, “mental health is [not] a priority,” and that help-seeking often wasn’t in “the form of . . . therapy . . . [as] it is in the Western world” (YP2). YPW spoke of feeling “torn between different cultures” due to having “different influences and different expectations culturally” (YP5), for example, the expectations in Pakistani culture not to “admit to someone that you were suffering or even seeing a counsellor” (Parent2), compared with the British culture of having more open conversations about SMHPs.

Most participants discussed Pakistan to be a private society with a culture of “keep[ing] [mental illness] behind closed doors” (YP1), and that there is “very much a sense of secrecy that you shouldn’t tell people your weaknesses because there’s such a big sense of community . . . they think it’s dangerous” (YP2). In line with this, participants shared concerns that disclosing SMHPs would result in negative social and professional consequences, some of which were gendered: “Their daughter gets labelled as somebody who’s got mental issues then she’s definitely not getting married” (Parent1). As such, all participants spoke of hearing stigmatizing views from the Pakistani community. Commonly these included narratives that mental health problems are “a weakness” (YP1), “a choice” (YP5) or mean you’re “crazy” (YP4).

Several participants also spoke of *fears of medication over prescription*, following experiences of health care in Pakistan where “people who do get mental health support always end up on medication” (YP6), which was perceived unnecessary. Participants described witnessing the “negative impacts [of medication] on people [they] really care for” (YP4), which further exacerbated fears. Participants worried about the same pattern in the United Kingdom, creating a reluctance to seek help in either place.

The strength of these cultural influences differed based on generation, with participants noting a *generational shift to more positive mental health views*. Participants spoke of how in older generations, mental health problems are “a taboo” (YP3) but that younger generations were becoming “more open to the reality of mental health (problems)” (YP2). There was also a narrative surrounding the “difference . . . around Pakistanis who are second, third generation” (YP6) compared with first-generation Pakistanis who were perceived as less open, which may relate to the differing cultural influences in their upbringing.

##### Religion When Distinct From Culture Promotes Help-Seeking

Participants discussed that *religion, when distinct from culture, promotes help-seeking* with narratives that Islam expressed “a really positive message” (YP2) with regards to mental illness. Many participants spoke of how “Islam encourages you . . . to reach your full potential” (Parent1) and was a source of support which facilitated help-seeking. In addition, one young woman spoke of how it was largely through her “detachment with religion” which was “internally . . . [her] biggest umm change” that highlighted her SMHP and need to seek support. Despite this, participants voiced how “religion and culture . . . [are] entwined in a way that they shouldn’t be” (YP1) and that this led to community views that they “need to speak to God and pray more” (YP6) if experiencing mental illness rather than seek professional help. Most participants spoke of a clear distinction between religion and culture voicing that “religion is not telling you not to access anything . . . it’s the culture” (Parent2), highlighting that when combined with culture, religion could instead become a barrier to seeking help.

##### Social Networks Influence Help-Seeking

All participants spoke of the influence of social networks in help-seeking for SMHPs. Both parents and YPW described both Pakistani and British *peers as supportive and accepting*, “provid[ing] some understanding . . . [and] a bit of a safety net around [them]” (YP1), which ultimately facilitated help-seeking. Many spoke of how peers “shaped [their] attitude” (YP5) and encouraged help-seeking. For some, peers directly initiated help and “told the school [they] were struggling” (YP2).

In contrast, the influence of family and academic settings was mixed. While YPW shared that **family support is valued**, they described how it was sometimes missing. For some, family was described to be “their support system” (YP4), and to “encourage [them] to go talk to somebody” (Parent1). Others, however, described not “feeling like [they] could talk to them” (YP2), due to family “minimis[ing] [SMHPs] for a long time” (YP5), stigmatizing SMHPs, or holding a strong sense that their child should have gratitude and “not act like [they’re] struggling when [their parents have] struggled more” (YP6).

Similarly, participants spoke of how *support in academic settings can be pivotal* but often lacking. During school, most YPW spoke of positive experiences of staff, with staff encouraging help-seeking and providing counseling. These “positive experience[s] of seeking help” helped YPW to “learn that . . . [help-seeking] could have good consequences” (YP1). This contrasted with the views of parents, however, with one describing school staff as “clueless” to the presence of SMHPs (Parent1). Furthermore, at university, YPW spoke of how [academic staff] will not accommodate you if you are not doing well” (YP4) with mental illness, resulting in YPW not sharing their difficulties with university staff due to concerns for their reputation.

Participants shared that *talking to people with lived experience is empowering and daunting*, depending on the narratives shared. Many YPW described hearing others speaking out as “admirable . . . and empowering” (YP1) in their own help-seeking journey. At times, however, participants described the experiences others shared “were not great, so that was a bit daunting” (YP3) such as experiences of racial discrimination. These negative experiences could lead to YPW developing prejudices of help-seeking that were based on “secondhand experience as opposed to [their] own” (YP6).

##### Education and Awareness Surrounding Mental Health Problems Matters

Both YPW and parents spoke of how *good MHL is necessary for help-seeking*. Most participants spoke of delays in realizing they or their child were experiencing SMHPs due to low MHL: “I’ve had a lot of problems trying to deal with my anxiety from a young age, but I didn’t really understand that that was what was the issue” (YP5). For many YPW, SMHPs were often misinterpreted which could result in self-stigma: “I was just kind of low and I was like oh I’m just being really difficult and problematic” (YP6).

Both YPW and parents spoke to the *importance of open conversations about mental health* to help gain perspective and encourage help-seeking: “Talking about it, like making it seem normal, was a big umm like motivator for me to even start thinking about seeking therapy” (YP5). One young person spoke about how “there’s a specific way that [these conversations] need to take place,” however, due to concerns that SMHPs may become trivialized among peers depending on their narrative.

Many participants flagged that *education about mental health problems and services is powerful*. YPW spoke of the importance of additional, and earlier education in schools and through social media. Both parents and YPW emphasized the importance of parental education and “upskilling . . . actually preparing the parents from the South Asian families to have groups . . . where they discuss these issues” (Parent2): **“**We also have to equip . . . the parents . . . who are stuck still in that point of view that mental health is not something we talk about” (YP1).

##### Internalized Stigma Hinders Help-Seeking

The experiences of multiple cultural influences, social networks, and education and awareness (or lack thereof) could contribute to an internalization of stigma in YPW, leading to *negative views of mental health problems and help-seeking*, which discouraged sharing with social networks and having open conversations about mental health problems. Participants described how these views were largely determined by their upbringing which in some cases could hinder help-seeking: “I think I had a pretty negative view of umm help seeking. Umm and I probably still do to an extent. Just because it was never something that was supported culturally with my family.” (YP5). These views could result in “never fully liv[ing] down the stigma, even in [their] own head” (YP4).

YPW commonly shared *fears of the community response* if they were to disclose SMHPs, with worries of what their friends and “family might do or think if they find out.” (YP1). Some spoke of how this fear restricted disclosure even within mental health settings: “I also couldn’t speak about everything because I knew . . . if you’re a danger to yourself we’re going to have to tell your parents and I didn’t want my parents to know” (YP2). This demonstrates how beliefs about the self and others directly influenced help-seeking.

Finally, YPW expressed *worries about future professional reputation* if they disclosed SMHPs. Some YPW spoke of not wanting to share SMHPs due to fears it would affect their academic work, their jobs, and how they were perceived professionally: “If you go to the NHS and if they decide to put a label on you . . . that’s always gonna be carried on you, and that can implicate you” (YP6). “It will impact how high performing they see me” (YP4).

#### Service-Level

##### Systemic Issues Hinder Access to Health care

YPW spoke of finding *systems difficult to navigate* in the United Kingdom and requested greater outreach and information: “I’m not sure . . . how I would find somebody that I would connect with or be able to understand the issues that I have” (YP5). “The outreach element is really important . . . getting it out into the community” (YP1). Participants spoke of several different first contacts, and challenges in referrals from one service to the next. One participant spoke of an additional “barrier in Oxford where people (students) are quite kind of transitory in their [nature]” (YP6), making it particularly challenging to access professional help.

YPW shared that *service pressures reduced accessibility* of health care, such as “the waiting list to actually get someone [being] extremely long” (YP3), inability to offer treatments, and limited sessions: “I was constantly told during the session like that’s beyond my remit, like we’re here to talk about da da” (YP6). One parent also highlighted that “when you have a secure relationship with somebody (e.g., GPs), it’s easier to speak” but due to service pressures that’s “changed a lot and it makes it harder” (Parent2).

##### Culturally Informed Services Are Important

YPW highlighted the need for culturally informed services, and how y. For example, one participant spoke of a professional who asked, “could you explain that a little bit?” or you know umm “what does that mean to you?” and shared how “those little things make you feel heard . . . so that . . . the cultural part of your experiences . . . isn’t ignored.” (YP1). Many participants, however, described that staff lacked awareness, and at times, interest in their culture: “I felt like she didn’t even want to hear about those things.” (YP6). Participants were eager for greater cultural awareness among staff and suggested better assessment questions that included an “awareness of the differences in British culture, and Pakistani culture” (YP5).

YPW also highlighted that *care that matches their preferences is ideal*, which was mirrored by parents. Participants highlighted feeling “more comfortable speaking to a woman” (YP4) and younger professionals due to “associat[ing] somebody who is older with having patronising attitudes to mental health” (YP5). Some YPW also highlighted a preference for staff of similar ethnicity. A choice in how care is received was emphasized including “a more anonymous experience” (YP6), and options for peer support throughout the process. It was shared that “having someone even in the referral process that you identify with that will . . . not just understand but have . . . somewhat experienced what it’s like to be in that situation” (YP2) would be particularly beneficial in fostering a more comforting journey to help-seeking where YPW could feel more understood.

## Discussion

We set out to preliminarily explore how YPW living in the United Kingdom, and their parents, make the decision to seek help for SMHPs and their perceived barriers and facilitators to this. Help-seeking was informed by YPW’s own beliefs and internalized stigma, which were shaped by the interplay between Pakistani and British culture, social networks, and mental health awareness. Religion was found to be distinct from culture and to facilitate help-seeking. Participants highlighted that both the structure and cultural context of services impacted access to professional help, and recommended both individual- (e.g., increased education and awareness) and service-level implementations (e.g., greater choice over care) to facilitate accessibility of services.

The results of this study highlight the challenges YPW face navigating the relationships between Pakistani and British cultures in relation to help-seeking for SMHPs. Both YPW and their parents spoke of stigmatizing views toward mental illness within Pakistani culture, consistent with previous research (Bonanno et al., 2021; Sangar & Howe, 2021). Many participants discussed a generational shift to less stigmatizing views toward mental illness, both in younger ages but also in later generations of Pakistani immigrants. This may be explained by prior research suggesting that first-generation foreign-born children are more likely to relate to their country of origin (Close et al., 2016; Phinney et al., 2000), a country which participants spoke of as holding high mental health stigma. Finally, all participants who discussed religion highlighted this as a source of support in help-seeking. This is consistent with one recent qualitative study in Pakistanis which found religion to be an effective source of coping and a therapeutic resource (Ayub & Macaulay, 2023). Most earlier research, however, has shown cultural perceptions that mental health problems are a result of religion-related “black magic” and therefore something not to seek mental health support for, still exist (Ali et al., 2017; Cinnirella & Loewenthal, 1999). This study highlights a need for greater understanding of the contributions of religion and culture as distinct when understanding processes for help-seeking for SMHPs.

This study found that cultural influences were strongly linked to internalized stigma and worries about personal reputation, findings that have been previously demonstrated in Pakistani communities (Ali et al., 2017; Husain et al., 2020; Shefer et al., 2012). Feeding into this internalized stigma were the influences of social networks and education (or lack thereof). Notably, participants spoke of how other young people most often reduced this stigma, while experiences with family and academic settings was more mixed. This self-stigma was exacerbated by a lack of awareness and understanding of SMHPs. Interestingly, despite the expected importance of intersectionality of sex and ethnicity, YPW and their parents rarely spoke of barriers and experiences related to being a woman, or the influences of patriarchy, but rather related these solely to their ethnicity and cultural background. It is possible this may relate to the specific demographic of this sample, as all young women interviewed shared, they were living away from family in affluent U.K. cities and spoke of being well-educated in U.K. universities. As a result, they may have experienced fewer of the cultural restrictions that YPW in the literature have typically been reported to face. Further research is needed in a sample with broader demographics to ascertain this. Alternatively, issues related to gender norms and “izzat” identified elsewhere in the literature may be less prevalent in this particular community in Oxfordshire. These findings underscore the importance of not making assumptions about the impact of cultural values around gender and sex, and the need for clinicians and researchers alike to remain open-minded and nuanced in their exploration of barriers to help-seeking.

Many participants spoke of the power of education in facilitating help-seeking. This was raised as particularly important for parents, whose knowledge is often required to identify SMHPs and access appropriate services (Tully et al., 2019) and may be especially reduced in this population where parents may not have been raised in U.K. culture. Previous research has highlighted a need for MHL education programs for parents (Hurley et al., 2020), however, there is little evidence to suggest that current methods for this are effective (Peyton et al., 2022). In this study, parents suggested the use of community-based groups to improve MHL, highlighting the importance of PPI in these initiatives.

Alongside these individual barriers to help-seeking, participants spoke of several service level barriers such as difficulties navigating services and limited remit. These findings are consistent with those of previous systematic reviews that explore barriers to help-seeking in young people in the United Kingdom (Radez et al., 2021; Salaheddin & Mason, 2016). In addition, this study highlighted service-level barriers underpinned by their cultural values such as lack of professionals whom they felt able to relate to, with most stating a preference for young, female professionals.

The findings of this study provide clear clinical and policy-related implications. Chiefly, almost all participants emphasized a need for greater focus on their cultural experiences in health care. Prior policy has focused on increasing professionals’ cultural competence in line with equality, diversity and inclusion initiatives in the NHS (NHS England, 2022a). More recent research, however, has suggested moving to a model of cultural pragmatism. While this still involves clinician’s developing and implementing skills in cultural competence, (such as greater understanding of the nuances between religion and culture), there is a greater emphasis on appreciating that both clinical and cultural understanding can be held true simultaneously, without one taking precedent over the other (Yahalom & Hamilton, 2023). In this light, while increasing MHL in Pakistani communities still remains of value, the goal is not to shift patients to the clinician’s way of thinking about mental health and interventions which may be influenced by their assumptions of the client’s culture. Instead, this approach moves away from a “one size fits all” approach and emphasizes understanding the individual’s experience of culture, *how the person’s views and behaviors may be functional within their specific cultural contexts*, and taking time to explore how this can work alongside the clinician’s understanding and experience of mental health when implementing interventions. This may be of particular importance when working with YPW who may hold multiple cultures and intersectionalities.

Wider systemic and service implications were also indicated. Participants voiced a desire for a choice of health care professionals, with most requesting younger, female professionals, in line with their cultural values and beliefs that younger generations would be less stigmatizing. YPW also requested the support of someone who understood their cultural context throughout the referral process. Previous research in the Netherlands has found that the inclusion of young peer support workers (PSWs) in services can be effective in reducing disparities, promote hope, and facilitate engagement (De Beer et al., 2023). In the United Kingdom, PSWs have been introduced as a new NHS workforce (Lawton-Smith, 2013), however, to date this has solely focused on adult populations. Based on the findings of this study, the inclusion of young PSWs with cultural awareness may be an area for development. Finally, both mental health services and educational institutions can improve help-seeking through raising awareness of SMHP and providing better understanding of how to access services. Participants suggested this may be particularly beneficial if done through social media and community outreach, and that this may be particularly important for parents of YPW.

There are several limitations. First, the study includes a small number of participants, particularly parents and second-generation migrants, and although this data was not explicitly collected, all participants spoke of being in university or having university-level education. This meant that only a limited number of views were heard with a small range of demographics, reducing Generalizability of the findings, and making it challenging to explore further nuances such as differences in experiences based on generation or education. Furthermore, information regarding social class, birth order and family history of mental health conditions was not collected, and is recommended for future research to promote understanding of how these factors may influence help-seeking. Several imposter participants were recruited as part of the study, however, the data from these participants were not included in the paper. It is likely this related to remunerations provided for taking part, therefore, future studies may wish to not advertise remunerations, and to follow the guidance provided by Ridge et al. (2023) such as checking email configurations. In addition, all participants had sought mental health support, therefore findings do not reflect the barriers of those who have not yet accessed professional help. Given this, qualitative research focusing on these YPW who have not yet been able or willing to seek help for their SMHP is a key priority. These limitations may reflect the heightened stigma within this community in speaking about SMHPs, in addition to accessibility issues of the research due to most researchers being White British and, and exclusion criteria (e.g., insufficient English for interviews) that may have paralleled the barriers YPW and their parents face in accessing services. We recommend that future studies facilitate recruitment using community outreach from those in the Pakistani community, as well as using interpreters so that the voices of more YPW and parents can be heard.

## Conclusion

This preliminary study highlights the complexities of help-seeking for YPW experiencing SMHP. The interplay of Pakistani and British culture, and internalized stigma were prominent influencing factors on the decision and ability to seek help. Recommendations given by YPW, and their parents largely focused on increased education and awareness through schools and community outreach, in addition to providing more culturally informed services with greater cultural awareness and choice over care. These findings highlight the need to for further research to understand the specific barriers experienced by YPW to inform the design of more inclusive services. Further research would also benefit on testing the suggestions of YPW and their parents such as the integration of young peer workers into services.
